# Extracts from a Paper on the Prevention of Insanity

**Published:** 1883-09-20

**Authors:** Nathan Allen

**Affiliations:** Lowell, Mass.


### EXTRACTS FROM A PAPER ON THE PREVENTION OF INSANITY.

BY NATHAN ALLEN, M.D., OF LOWELL, MASS.

In all the discussions on insanity found in reports, journals and books, there is scarcely a reference to prevention, till within a few years. The most decided statement appeared in the 17th annual report of the Commissioners in Lunacy for Scotland. This is so much to the point that I am induced to make the following quotations:

"It is impossible to come to any other opinion than that insanity is, to a large extent, a preventable malady; and it appears to us that it is in the direction of preventing its occurrence, and not through the creation of institutions for its treatment, that any sensible diminution can be effected in its amount. Lunacy is always attended with some bodily defect or disorder, of which it may be regarded as one of the expressions or symptoms.

"We must, therefore, attempt to prevent its occurrence in the same way as we attempt to prevent the occurrence of what are called ordinary bodily diseases; and if it be admitted that, to a large extent, preventable diseases exist among us in consequence of this ignarance of the people, it is clear that we can only convert the preventable into the prevented by the removal of that ignorance through a sounder education. Men must be taught that it is their duty, and not merely their interest, to understand the laws of health and to make them eventually the rule of their conduct. In short, we can only hope that preventable insanity, like other preventable diseases, will be diminished in amount when the education of men is so conducted as to render them both intelligent and dutiful guardians of their own physical, intellectual and moral health.

"To this, and not to any machinery, however good it may be, for the cure and treatment of insanity which has actually arisen, can we reasonably look for a diminution in its amount."

No higher testimony on this subject could be quoted than that from the Lunacy Commissioners of Scotland. Several distinct points are here brought out: 1st. That insanity is a disease, and can be prevented, as other diseases are. 2d. For this purpose similar means must be used as are employed to prevent diseases generally. 3d. The public must be better educated and trained in respect to the laws of health. 4th. By this process only can we expect a diminution of the disease. Lunatic hospitals alone will never do it.

*Hereditary Influences.*—No fact connected with insanity is more firmly established than that it largely originates directly from inherited tendencies; and, if we include all weaknesses, imperfections and diseases rising from the same source, it may be found that more than half the insanity of the present day can be traced directly or indirectly back to hereditary sources. By careful study and observation it is not difficult to discover the physical differences und hereditary tendencies in the families here described.

Let it be understood, more and more, that disease and insanity come mainly from inherited causes; let young men and women become thoroughly acquainted with such facts, and it must lead to greater carefulness in forming matrimonial alliances.

When the community is generally informed on this subject, inquiries will at once be made as to the health, the constitution, and the inherited tendencies of candidates for marriage. Such inquiries are already made in a quiet way, and they must increase, in the very nature of things.

In the prevention of disease and insanity, then, heredity has a powerful influence.

There is such a thing as a normal standard of physiology, where the structure and functions of all the organs of the body, including the brain, are well-nigh perfect. With such organizations to start with in life, how would disease and insanity diminish! And just in proportion as we find organizations approximating to this standard, do we find less disease of body and mind. It is true, the environment, the circumstances, the habits and employments of individuals, have a powerful influence on their health and mental development. All these things may unfavorably affect, to some extent, those having naturally the best organizations; but their effects would not compare, in extent or magnitude, with the evils growing out of weak, diseased and ill-balanced organizations.

The fault does not arise merely from the want of soundness in structure or health of function; but often from the want of balance and harmony in action. This is the starting-point, not only of a great deal of disease, but of much mental derangement. If there could be a more perfect balance or harmony in the exercise of all parts of the body, including the brain, it would prevent a vast amount of disease and insanity. It might take two or three generations to bring about these changes, but they would assuredly come, provided the proper means were used. Such changes would constitute a radical and permanent improvement.

*Causes of Insanity.*—In the last quotation from Sir James Coxe, is a summary of the primary causes of insanity, from one who had made the subject a special study for over twenty years. Says Sir James, the leading factors are "dissipation in its various forms, overwork, meagre fare, lack of ventilation and neglect of moral culture." It will be seen that each one of these covers a great deal of ground. Passing by the last point—neglect of moral culture—the other four constitute the chief sources of disease of all kinds, some of which terminate in mental derangement. But nearly all these great agencies, productive of so much disease of body and mind, are subject to human control, and can be more or less checked, if not entirely prevented.

The first named, dissipation, is a fruitful source of insanity. This may consist in drinking habits, in the use of tobacco and opiates, or in the abuse of the sexual organs by licentiousness and solitary vice. These evils are all the results of voluntary acts, the work of a free agent, and so they can be prevented.

Overwork of body or mind not infrequently brings on mental derangement.

Meagre fare and bad air are evils which multitudes of poor people cannot always escape. Neglect of moral culture is an evil directly connected with the choice of individuals, and the state of public morals. It is a sin or an evil which can be corrected, wherever the fault may be, and there certainly can be no necessity or justification for any neglect. Dr. Henry Maudsley, the distinguished foreign alienist, spoke on this point as follows:

"It is to the perfecting of mankind by the thorough application of a true system of education that we must look for the development of the knowledge and the power of self-restraint, which shall enable them, not only to protect themselves from much insanity in one generation, but to check the propagation of it from generation to generation. Unhappily, we are not yet agreed as to what should be the true aim and character of education. Regarding the subject from a scientific point of view, the best education would seem to be that which was directed to teach man to understand himself, and to understand the nature which surrounds him, and of which he is a part and a product, so as to enable him, as its conscious minister and interpreter, to bring himself into harmony with nature in his thoughts and actions, so to promote the progressing evolution of nature through him, its conscious self."

Dr. Maudsley, here speaking of "perfecting mankind," says that it cannot be done till we have a "true system of education." The only way it can be done is through the body and the brain, and to do it we must also have some standard before us, some guiding principle to aid us. As to the "propagation of insanity" by hereditary influences, how can we understand the laws of inheritance unless we have some standard in physiology? When the laws of this science are fully understood, it will be found that the most powerful agencies for preventing insanity lie in this direction.

Again: it is well understood that the most favorable time to cure insanity is in its first stages; on this account, it is constantly urged that all insane persons, just as soon as any marked symptoms of the disease appear, should at once be sent to a lunatic hospital. This counsel has generally prevailed in acute and violent cases, but in the milder forms of the disease the friends frequently object and delay. It is a great step to take; there are certain forms of law which must be complied with; then, the dread of its effects on the patient, the trouble attending the removal, and the anxiety about the situation and treatment of the patient in the hospital, etc.: all these things cause delay, sometimes for weeks and months, and may prevent the patient from going till the acute stages of the disease are passed. The complaint is often made by superintendents that large numbers are sent to the hospital who cannot be cured, because they come too late. This is given as one of the reasons why the rate of cures is so small; for, taking all admitted into our hospitals, only about forty per cent., on an average, actually recover.—*Psychological Journal.*

